# Identification of differentially expressed genes, pathways, and immune infiltration in diabetes

**DOI:** 10.1016/j.clinsp.2024.100436

**Published:** 2024-08-02

**Authors:** Ying Liang, ShuXiang Wei, Xing Peng, QiLing Feng, LingLing Li, DieFei Liang, HongShi Wu, XiaoYun Zhang, ChuLin Huang, YongQing Lin

**Affiliations:** a Department of Endocrinology, Sun Yat-sen Memorial Hospital, Sun Yat-sen University, Guangzhou City, Guangdong Province, China; b Department of Endocrinology, Guangdong Tongjiang Hospital, Foshan City, Guangdong Province, China; c Department of Cardiology, Sun Yat-sen Memorial Hospital, Sun Yat-sen University, Guangzhou City, Guangdong Province, China

**Keywords:** Type 2 Diabetes, Differentially Expressed Genes, Bioinformatics, Hub Genes, Immune Infiltration

## Abstract

This study aimed to perform exhaustive bioinformatic analysis by using GSE29221 micro-array maps obtained from healthy controls and Type 2 Diabetes (T2DM) patients. Raw data are downloaded from the Gene Expression Omnibus database and processed by the limma package in R software to identify Differentially Expressed Genes (DEGs). Gene ontology functional analysis and Kyoto Gene Encyclopedia and Genome Pathway analysis are performed to determine the biological functions and pathways of DEGs. A protein interaction network is constructed using the STRING database and Cytoscape software to identify key genes. Finally, immune infiltration analysis is performed using the Cibersort method. This study has implications for understanding the underlying molecular mechanism of T2DM and provides potential targets for further research.

## Highlights

• GO enrichment analysis include CC, BP and MF in diabetes.
• Most significantly enriched pathways include thermogenesis, oxidative phosphorylation, and chemical carcinogenesis-reactive oxygen species.
• The proportion of macrophages M2, CDB T-cells, monocytes, and resting mast cells (M2, T-cells CDB, Monocytes, Mast Cells resting) in samples from T2DM patients is higher and more abundant in diabetes.
• The content of T-cells, testing NK cells, naive B-cells, and naive CD4 T-cells is relatively low in diabetes.

## Introduction

About 415 million people worldwide have diabetes, with a prevalence rate of 8.8 %. By 2040, about 642 million people are estimated to suffer from diabetes.^1^ Among them, Type 2 Diabetes (T2DM) is the main type of diabetes, taking about 90 % of diabetic cases.^2^ It is generally known that the molecular mechanisms of disease development generally refer to processes characterized by protein abnormalities due to genetic variation in DNA. In this case, T2DM is a complex disease involving the interaction of genetic and environmental factors.^3^ Thus, understanding the molecular mechanism of T2DM is crucial for its diagnosis and targeted therapy (Choi, 2010).

A striking molecular feature of T2DM is a reduced response to insulin, which is known as insulin resistance.^2^ In recent years, the main direction of many studies on the molecular characteristics of T2DM is insulin resistance (Zhao and Townsend, 2009; Stanford and Goodyear, 2014).^4^^,^^5^ However, in addition to insulin resistance, some studies have shown that metabolic disorders may be related to obesity caused by T2DM.^6^ Apart from that, studies have shown that changes in multiple genes and signaling pathways are involved in T2DM development.^7^ In this study, functional enrichment analysis of Differentially Expressed Genes (DEGs) in T2DM was performed, including gene ontology analysis and pathway analysis to explore the molecular mechanism of T2DM. In addition, infiltration refers to the infiltration of abnormal cells into human tissues or the appearance of body cells that should not appear under normal conditions, as well as the phenomenon that some diseased tissues expand to the surrounding, and abnormal substances or substances appear in cells or in the interstitium (Galon, Dieu-Nosjean and Tartour, et al., 2010). Excessive accumulation of some substances is also called infiltration. The study of immune infiltration has made a great contribution to the study of the disease, and the difference in the infiltration degree of different immune cells is highly correlated with the progression and prognosis of the disease.^8^^,^^9^

Bioinformatics is an interdisciplinary field that develops methods and software tools to translate biological data, in particular for large and complex datasets (Dixit and Ghanshyam, 2015). It can acquire, screen, evaluate, cluster, and validate known biological information, aiming to identify disease diagnostic markers and perform biologically meaningful annotations.^10^ A large number of genetic variants have been discovered and stored in databases, and bioinformatics analysis based on open-source databases has become more accurate and efficient (Mooney, 2005; Reimers and Carey, 2006). Therefore, this study will use bioinformatics analysis methods and databases to identify DEGs, biological pathways, proteins, and metabolites, screen and data analysis of highly expressed genes, and conduct interaction network analysis and immune analysis of key genes, so as to facilitate Follow-up studies on the pathogenesis of T2DM at the molecular level.

## Materials and methods

### Data selection

The Gene Expression Omnibus (GEO) database is a publicly available repository of gene expression datasets. The T2DM disease data for this study are derived from a micro-array dataset from the GEO database (https://www.ncbi.nlm.nih.gov/geo/). The relevant GSE series are screened according to the keyword “Type 2 Diabetes (T2DM)”. The expression matrix of GSE29221 and the micro-array data of the GPL6947 platform are selected as the research objects in this study. Using R to directly download GEO data and perform ID conversion.

### Identification of DEGs

After the data are downloaded and the preliminary processing is completed, this study uses R to identify DEGs, and completes the visualization of the DEGs of the dataset with volcano plots and heatmaps. Genes meeting cutoff criteria (p-value < 0.05 and |log fold change (logFC)| > 1.0) are designated as DEGs. After the DEG screening is completed, the gene information of 12 groups of T2DM patients and 12 groups of non-T2DM patients contained in the GSE29221 samples are grouped and divided into 12 experimental groups and 12 control groups. Use functions to obtain grouping information and examine the expression matrix of the sample. After the inspection, the DEGs are screened using the DESeq2 package in R, and a volcano plot is made for differential analysis and visualization to screen out DEGs between T2DM patients and non-patients.

### Functional enrichment analysis

Gene Ontology (GO) analysis and Kyoto Encyclopedia of Genes and Genomes (KEGG) pathway analysis and visualization are performed using R and clusterProfile. GO aims to make the functional description of gene products consistent in various databases, allowing the properties of gene products to be queried at different levels.^11^ KEGG is a knowledge base for systematic analysis of gene function, linking genomic information to higher-order functional information.^12^ In this paper, GO enrichment analysis is used to evaluate the Biological Processes (BP), Cellular Component (CC) and Molecular Function (MF) of DEGs (p < 0.05), and KEGG pathway is used to study molecular pathways.

### Protein-Protein interaction network (PPI) analysis and key gene identification

PPI network is a protein interaction involved in biological signal transmission, gene expression regulation, energy and substance metabolism, cell cycle regulation and other life processes. Its analysis contributes to the study of the molecular mechanism of disease from a systems perspective, discovering new drug targets, etc.^13^ STRING database (https://string-db.org) is an online resource for known and predicted PPI. In this study, the STRING database is used to establish the PPI network of DEGs and obtain the interaction network map.

Cytoscape is an open-source software platform for visualizing and integrating complex networks with various types of attribute data. This study used Cytoscape to visualize the interactions between proteins and identify hub genes.

### Immune infiltration analysis

Immune infiltration is a popular direction of tumor research. The difference in infiltration degree of different immune cells is highly correlated with tumor progression and prognosis.^14^, ^15^, ^16^ This method is applicable to the exploration of tumor diseases as well as non-tumor diseases. Through the expression matrix of highly expressed genes, the type and content of immune cells in mixed samples can be predicted. Common analysis methods include Estimate and Cibersort. In this study, the Cibersort method is used for analysis.

## Results

### Screening for DEGs

**Fig. 1:**
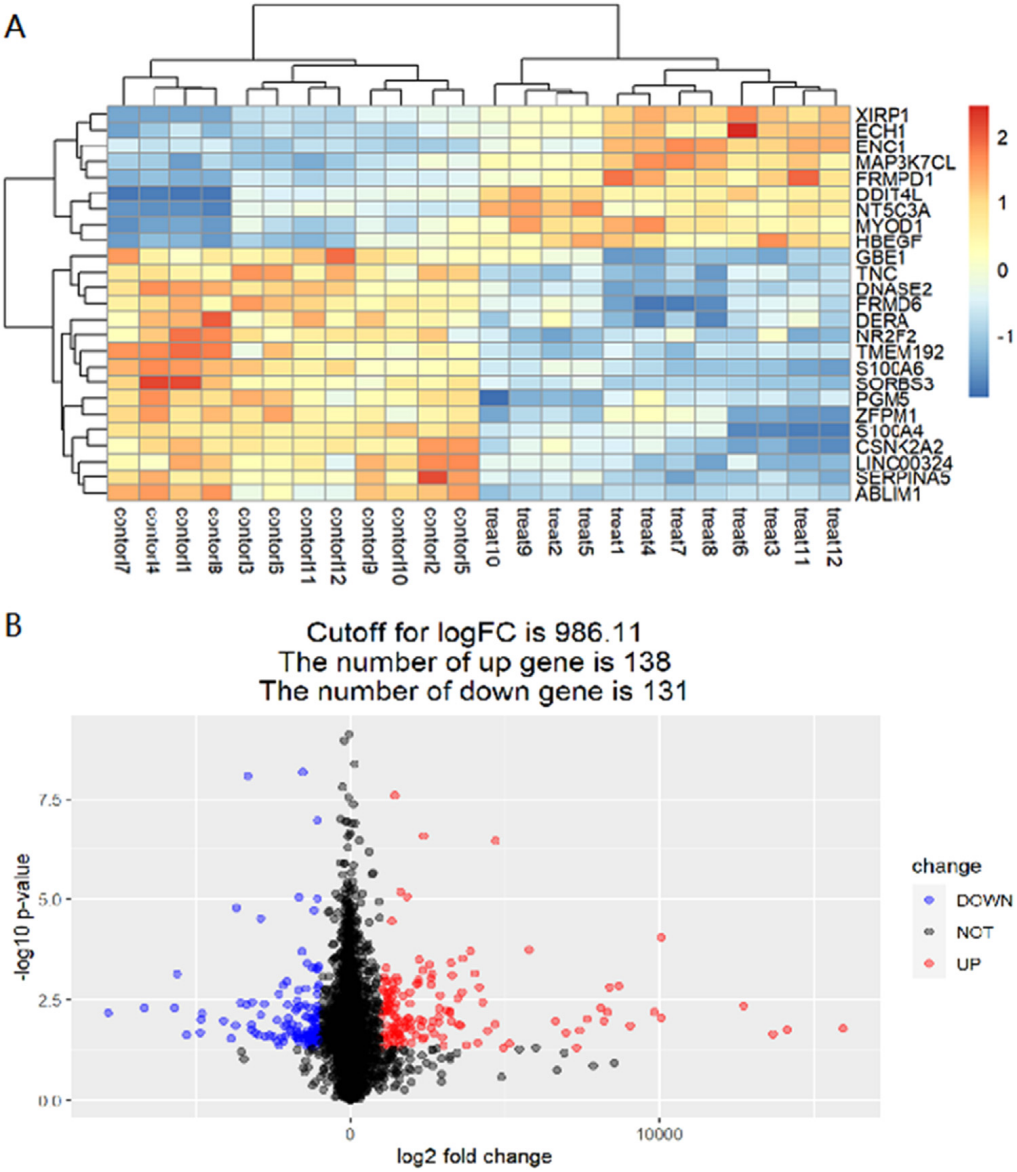
The distribution of differences in gene expression levels. Heat map of differentially expressed gene (A), the abscissa represents the sample, the ordinate represents the gene, red represents high expression while blue represents low expression, p < 0.05; Volcano plot of differentially expressed gene (B), the horizontal axis is the log2 fold change differential expression fold (according to the positive and negative log2 fold change value to judge whether the expression of these genes has increased or decreased), the gene with greater difference is distributed on the X axis at both ends; The ordinate is represented by -log10 p-value, and the p-value is converted to -log10.

Above all, this study collects T2DM disease-related microarray datasets from the GEO database and screened 19,217 DEGs by R. After that, the DEGs are screened according to the screening rule p-value < 0.05, |logFC| ≥ 2, and a total of 18948 genes with no significant difference and 269 genes with a significant difference are obtained. Among them, there are 138 up-regulated genes (red) and 131 down-regulated genes (blue). DEGs are displayed through a heat map (Fig. 1-A), the darker the color, the greater the degree of up-regulation or down-regulation. Significant and non-significant DEGs of the dataset are shown by making volcano plots (Fig. 1-B), where blue dots represent down-regulated genes, red dots represent up-regulated genes, and black dots represent genes with no significant differences.

### Functional enrichment analysis of DEGs

#### GO analysis

**Fig. 2:**
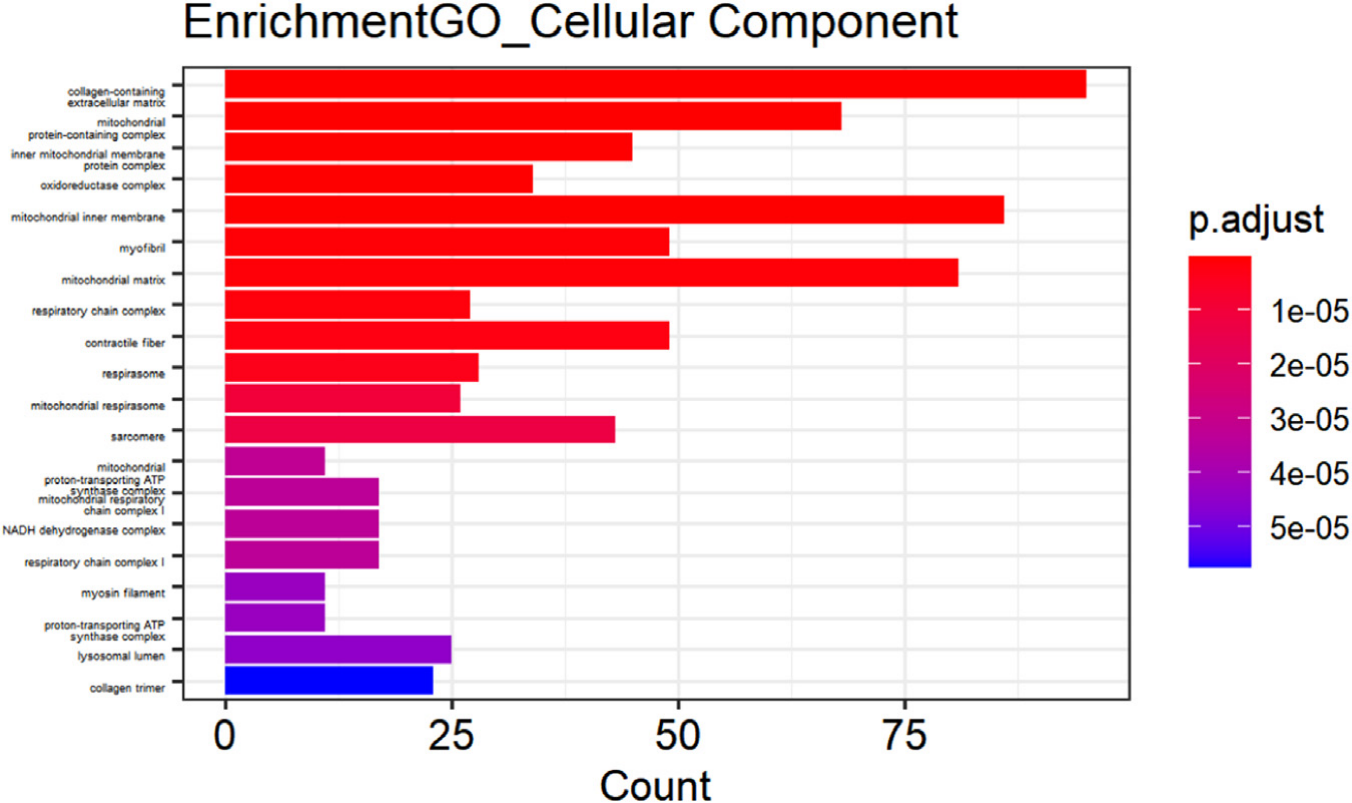
Cellular component of DEGs.

**Fig. 3:**
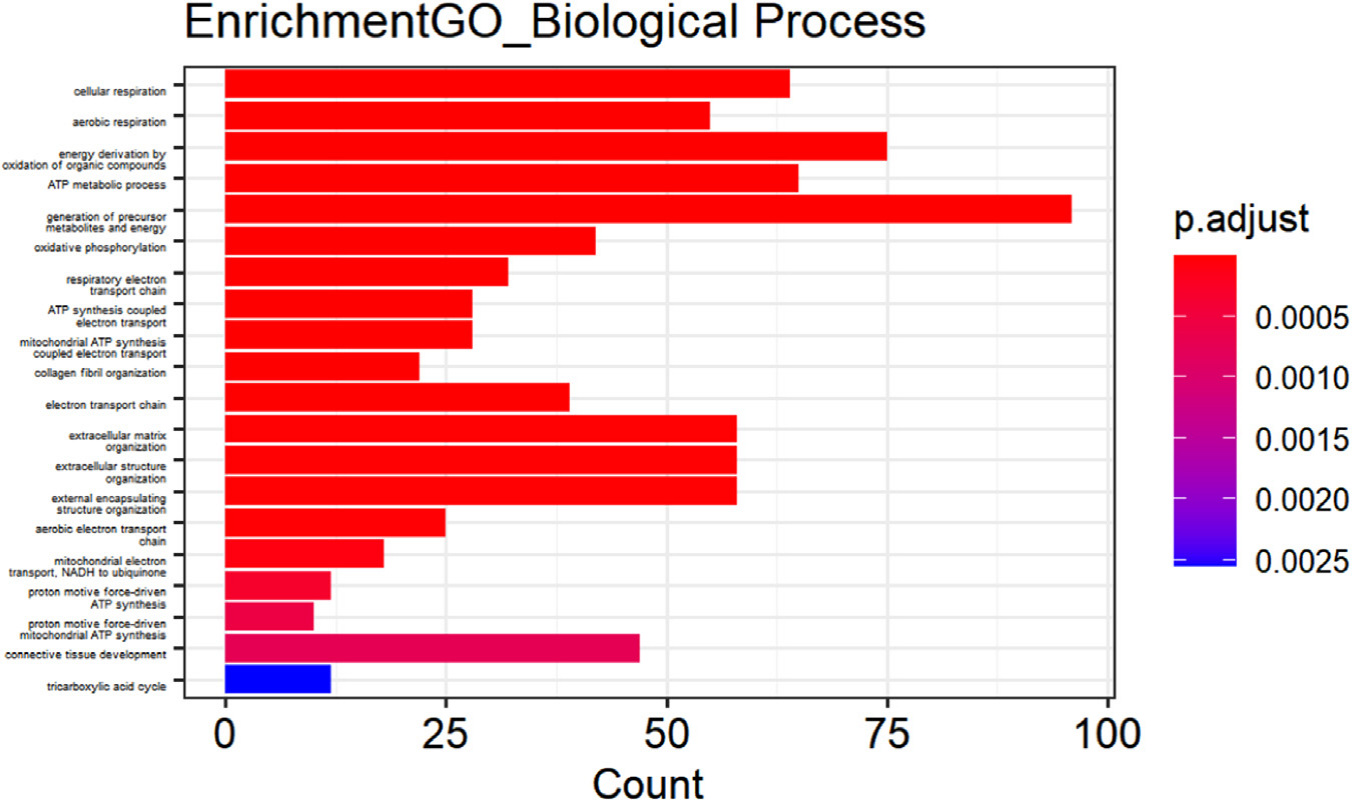
Biological processes of DEGs.

**Fig. 4:**
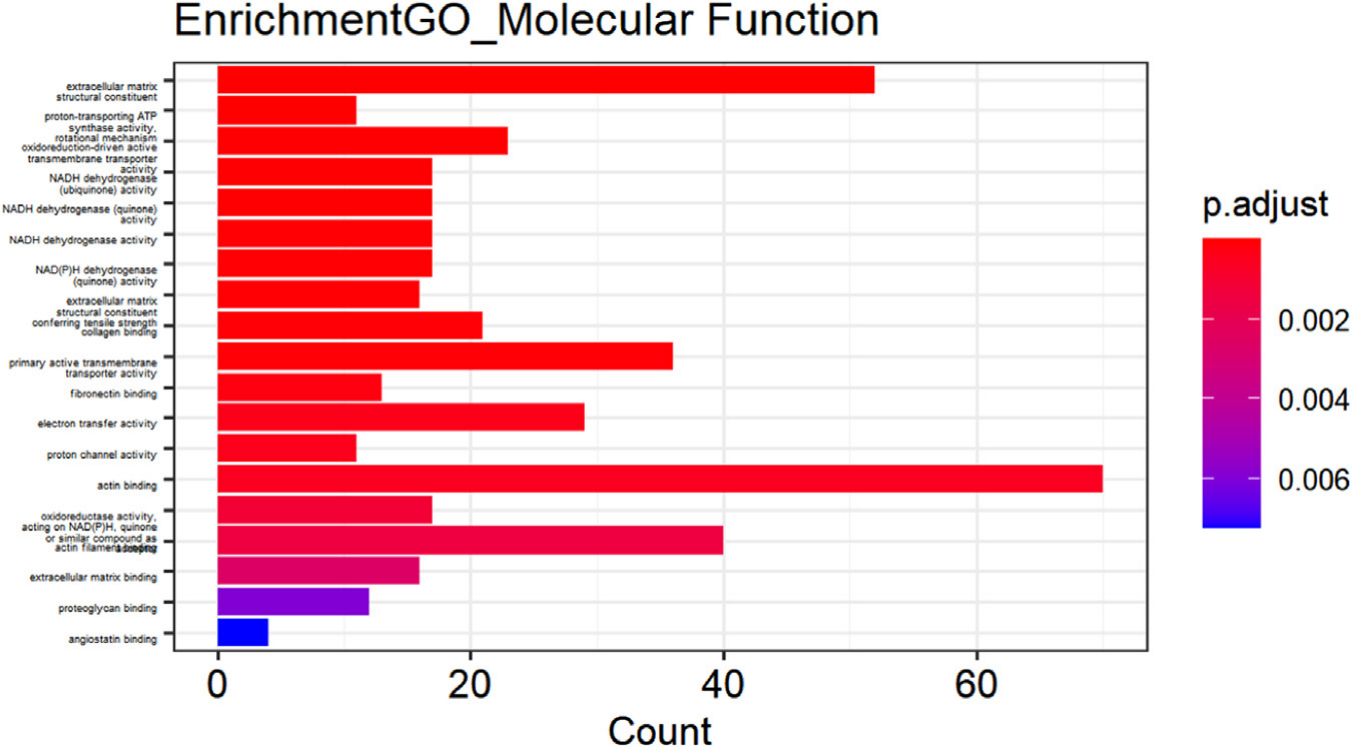
Molecular functions of DEGs.

Fig. 2 to Fig. 4 (ranked from small to large by p-value) show GO enrichment analysis, including CC (Fig. 2), BP (Fig. 3), and MF (Fig. 4). The expressed genes in this study are mainly involved in CCs including collagen-containing extracellular matrix, mitochondria-containing protein complexes, mitochondrial inner membrane protein complexes, oxidoreductase complexes, mitochondrial inner membrane, myofibrils, mitochondrial matrix, and respiratory chain. Complexes, contractile fibers, respiratory bodies, etc.; BF involved mainly include aerobic respiration, cellular respiration, energy derivation of organic compound oxidation, ATP metabolic process, production of precursor metabolites and energy, respiratory electron transport chain, oxidative phosphorylation, ATP synthesis coupled with electron transfer, mitochondrial ATP synthesis coupled with electron transfer, collagen fiber organization. Further analysis revealed that MF contains extracellular matrix structural components, proton transport ATP synthase activity, rotation mechanism, redox-driven active transmembrane transporter activity, NADH dehydrogenase (ubiquinone) activity, NADH dehydrogenase (quinone) activity, NADH dehydrogenase activity, NAD(P)H dehydrogenase (quinone) activity, extracellular matrix structural components that confer tensile strength, collagen binding and primary active transmembrane transporter activity.

#### KEGG pathway analysis

**Fig. 5:**
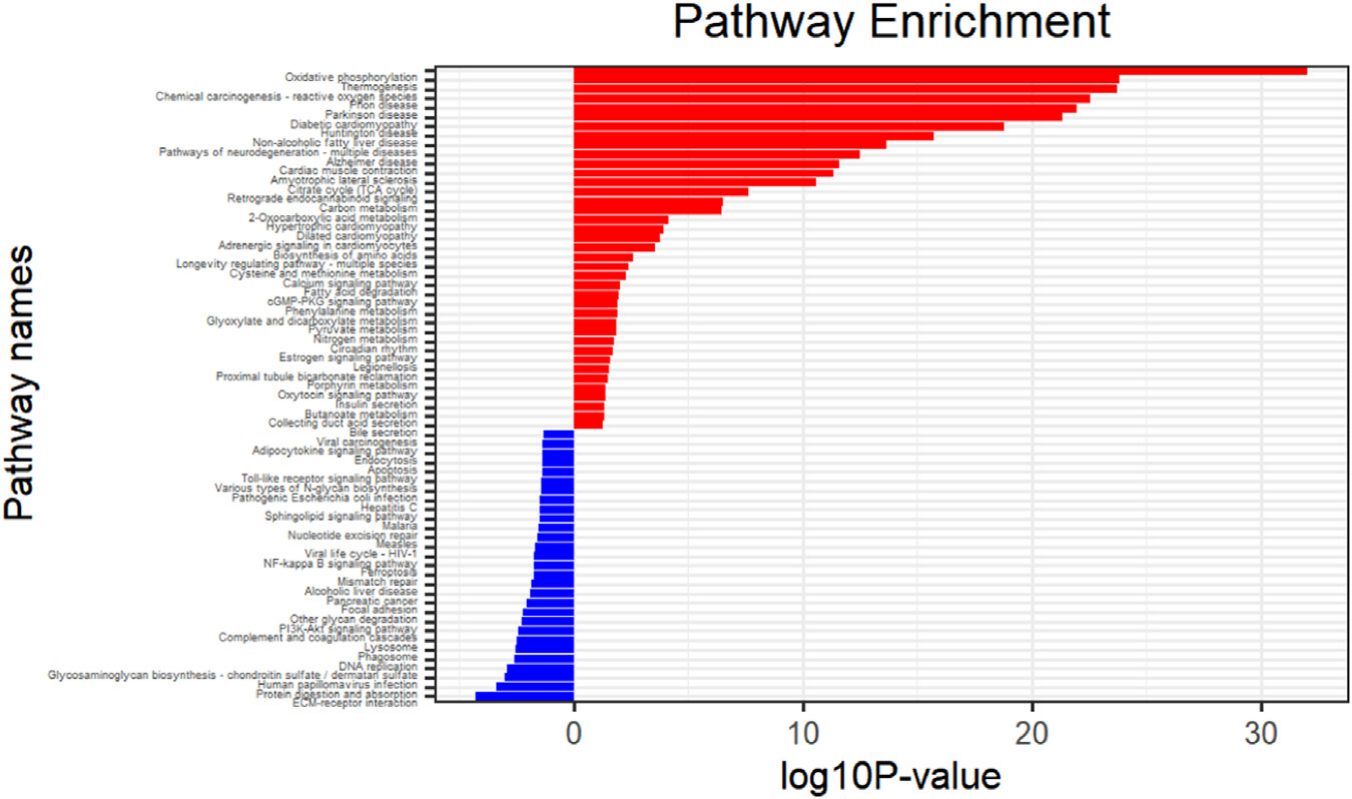
KEGG pathway analysis.

As Fig. 5 shows the most significantly enriched pathways are: thermogenesis, oxidative phosphorylation, chemical carcinogenesis-reactive oxygen species, prion disease, Parkinson's disease, and diabetic cardiomyopathy.

### PPI analysis results of DEGs

**Fig. 6:**
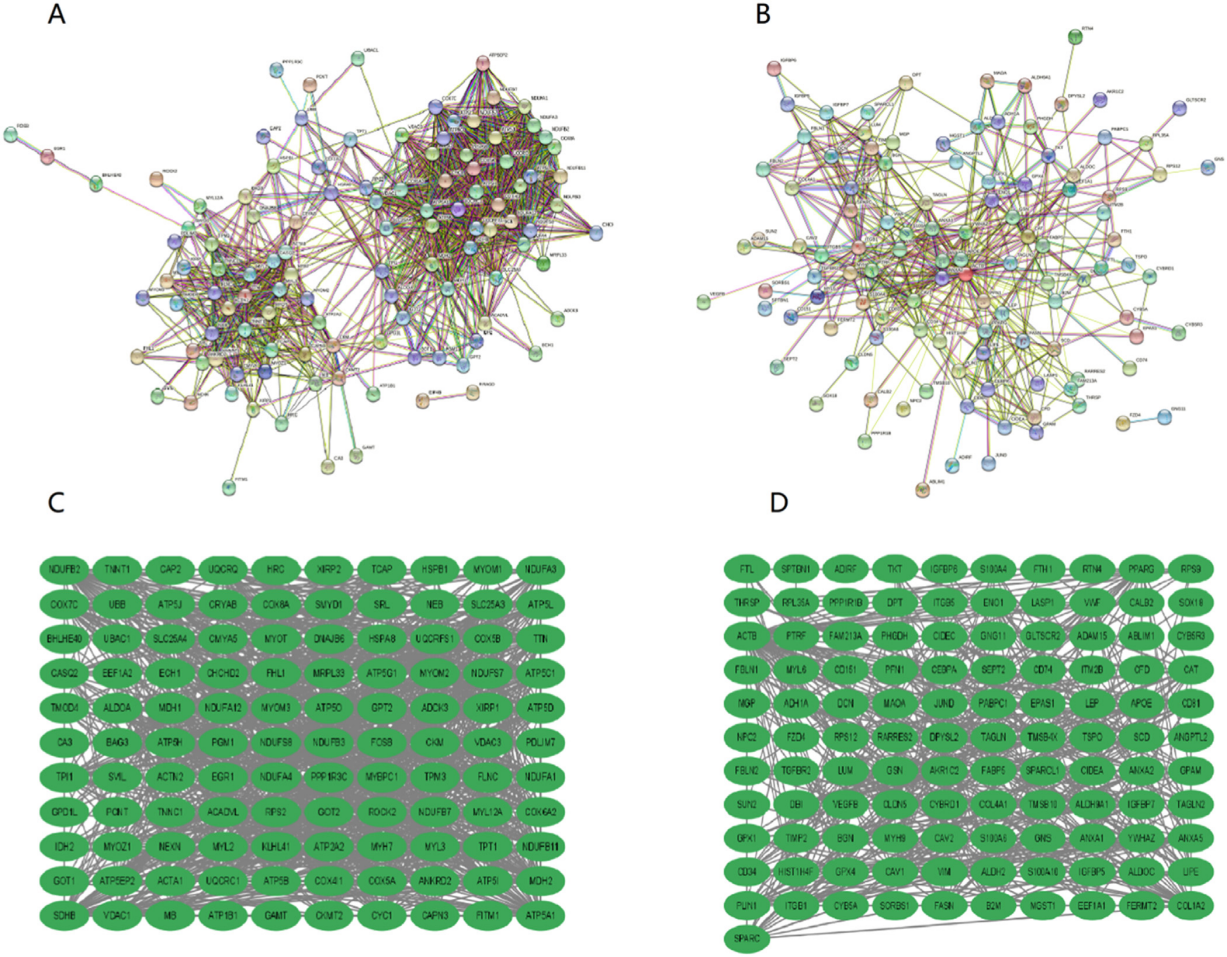
PPI plot of DEG. Differential protein interaction network diagram of up-regulated genes (A); The differential protein network diagram of up-regulated genes that can form an interaction relationship (C), and the single differential protein that cannot form an interaction relationship no longer appears; Differential protein interaction network diagram of down-regulated genes (B); The differential protein network diagram down-regulated genes that can form an interaction relationship (D), and the single differential protein that cannot form an interaction relationship no longer appears.

The PPI network of 269 DEGs is constructed by using the String online tool. After that, the entire PPI network is visualized by using Cytoscape software, and the tsv file, as well as the interaction network diagram, are obtained, as shown in Fig. 6 A and C on the left are the PPI diagrams of up-regulated genes, while B and D on the right are the PPI diagrams of down-regulated genes.

Tsv network file exported prior to import in Cytoscape software. Using the MCODE plug-in, the Betweenness Centrality (BC) method is used to screen out the top 10 key genes in up-regulated and down-regulated genes (Table 1).

**Table 1:** 10-hub genes screened by cytoscape.

Up regulated	Betweenness score	Downregulated	Betweenness score
ACTA1	1042.5537	ACTB	4511.061
COX6A2	1029.9016	PPARG	1019.9447
SLC25A4	908.635	ITGB1	958.7375
ATP5B	796.74066	CAT	835.65515
TTN	788.834	ENO1	735.9442
SMYD1	640.77625	APOE	640.5999
MYH7	603.8361	MYH9	461.29333
MYL3	591.0806	CAV1	447.75705
VDAC1	535.134	B2M	423.73395
MDH2	527.2745	COL1A2	418.40024

### Immune infiltration

**Fig. 7:**
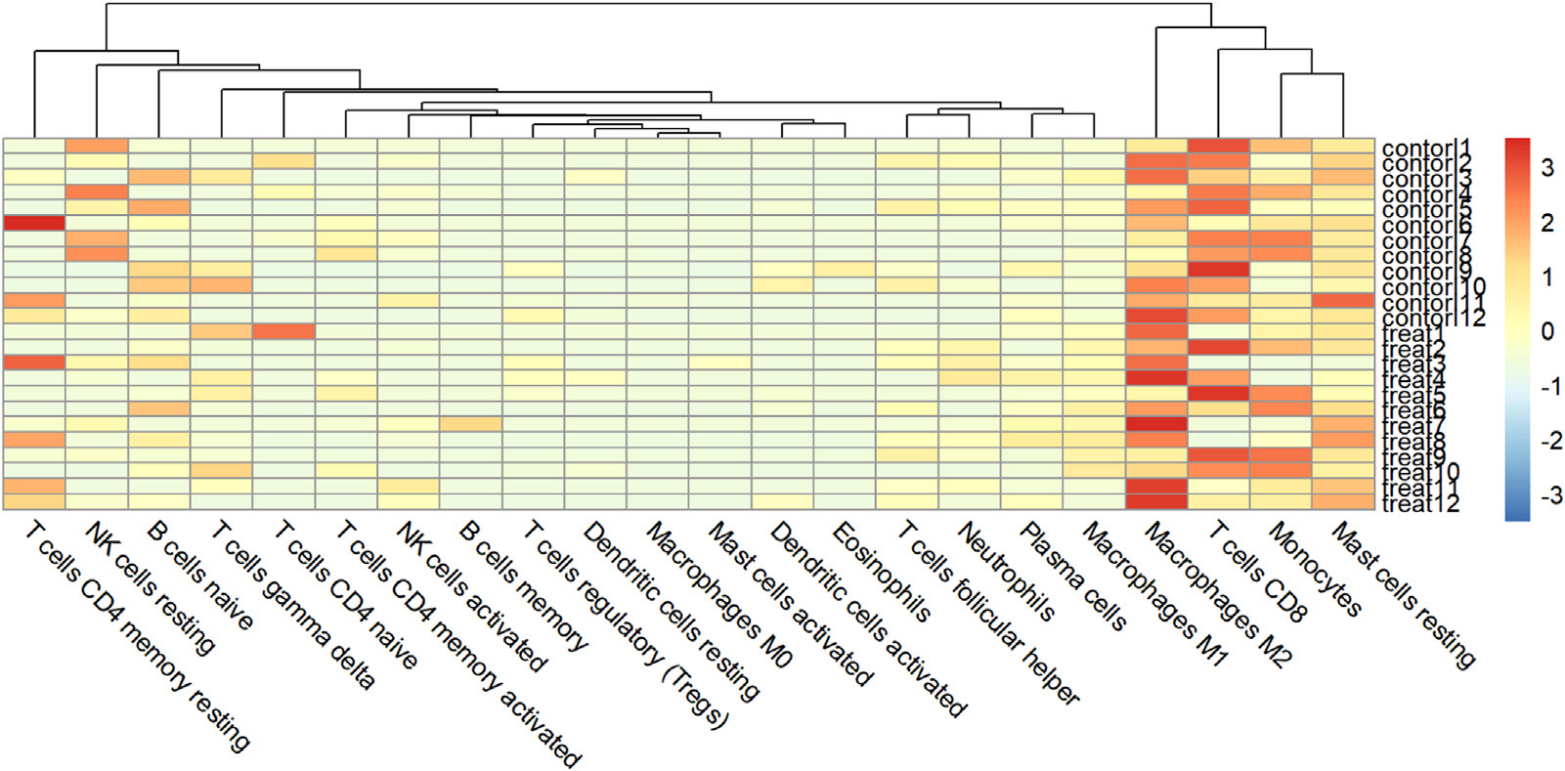
The relative proportion of 22 kinds of immune cells in the experimental group and the control group.

**Fig. 8:**
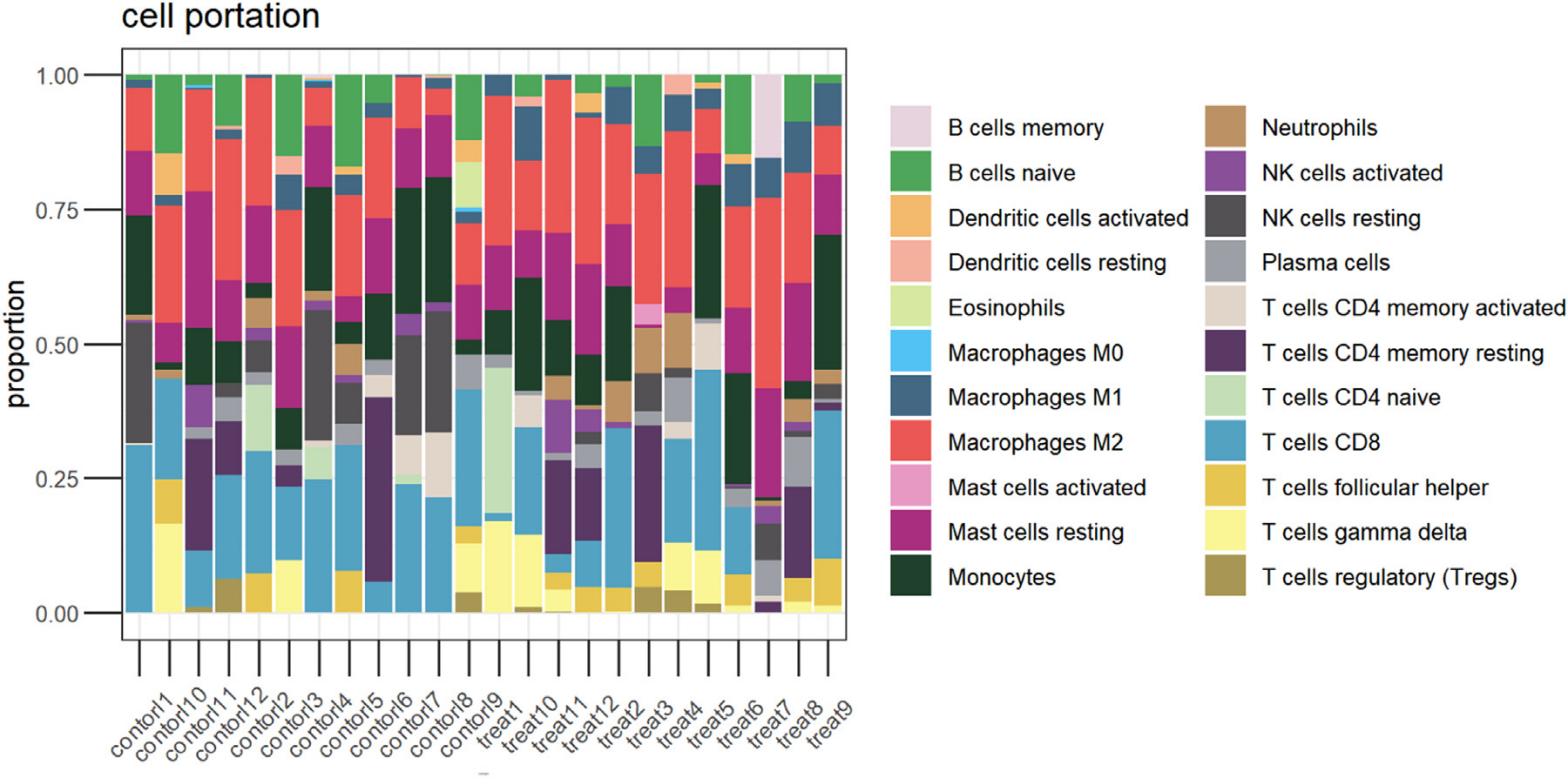
Expression and proportion of immune cells in each sample.

Immune infiltration analysis using the CIBERSORT method yielded the relative proportions of 22 immune cells in the two groups of samples (Fig. 7). The expression and proportion of immune cells in each sample are also analyzed (Fig. 8). The proportion of macrophages M2, CDB T-cells, monocytes, and resting mast cells (M2, T-cells CDB, Monocytes, Mast Cells resting) in samples from T2DM patients is higher and more abundant. However, the content of T-cells, testing NK cells, naive B-cells, and naive CD4 T-cells is relatively low.

## Discussion

There are some frontier studies for diabetes mellitus worth addressing prior to the discussion of these results. Above all, Eslami and Hemati believe that these significant changes in gut bacterial composition are related to both types of diabetes. A better understanding of the mechanisms related to the colon microbiome and glucose metabolism may provide innovative therapies to achieve optimal health of the desired gut ecosystem, thereby preventing and treating diabetes and related metabolic disorders. Besides, a recent study focused on the role of amino acid metabolomics in the treatment of type 2 diabetes and evaluated 21 amino acids whose metabolic disturbances are closely related to the pathogenesis of T2DM and related cardiovascular diseases (Alqudah, Wedyan, Qnais E, et al., 2021). These disordered amino acids can be therapeutic targets for T2DM. Furthermore, essential amino acids obtained from the diet and metabolized in adipose tissue and skeletal muscle, including branched-chain amino acids, ILe, Leu, and Val, are closely related to insulin resistance (Chen et al., 2016). In this context, Alqudah et al. found elevated Leu in T2DM patients, combined with two conclusions from the existing literature, the first being that inadequate energy intake and inactivity reduce skeletal muscle mass, leading to decreased muscle function and insulin resistance, which appears to cause skeletal muscle atrophy. The other is that exercise in essential amino acids, including leucine, stimulates protein muscle synthesis and growth (Drummond et al., 2009; Rudrappa et al., 2016). It can be inferred that combining exercise with increasing Leu levels may be a way to boost muscle protein synthesis and thus improve insulin sensitivity.

Differential expression analysis is usually the first step in bioinformatics analysis based on gene expression matrix, which helps us to observe the expression differences of genes in different samples, so as to determine the key genes and diseases for study. Commonly used gene expression data come from microarrays or high-throughput sequencing. The microarray data in this study are based on skeletal muscle tissue from 12 T2DM patients and 12 normal individuals. 29,432 genes are obtained through the GEO database and 269 significant DEGs are finally screened. Among them, there are 138 up-regulated genes and 131 down-regulated genes. GO enrichment analysis showed that DEGs played significant roles in BPs such as cellular respiration, metabolism, oxidative phosphorylation, and mitochondrial synthesis, which further revealed the possibility that T2MD is related to energy metabolism disorders. Pathway analysis showed that the DEGs are mainly distributed in oxidative phosphorylation, thermogenesis, chemical carcinogenesis-reactive oxygen species, prion disease, Parkinson's disease, and diabetic cardiomyopathy, which also indicated that T2MD is closely related to energy metabolism.

Additionally, 10 hub genes are screened out by PPI analysis of DEGs in T2DM, and further analysis of these genes and the proteins encoded by the genes is carried out below. Among them, ACTA1 encodes actin α1, while MYH7 and MYL3 are related to the encoding of myosin chains. In past studies, myosin and actin together have been implicated in overall cell motility.^17^ Thus, the key genes ACTA1, MYH7 and MYL3 reveal that T2DM patients exhibit abnormal cell motility. In addition, Cox6a2 is a respiratory chain gene, and studies have shown that Cox6a2-deficient mice can be protected from high-fat diet-induced obesity, insulin resistance, and glucose intolerance.^18^ In addition, fatty acid metabolism is important in regulating breast cancer progression,^19^ solute carrier family 27 member 4 (SLC27A4), as a fatty acid transporter, is also highly expressed in T2DM samples, and it is worthy of further study whether SLC27A4 can be used as a diagnosis of T2DM. Markers and therapeutic targets. The structure and function of the key gene ATP5B are closely related to mitochondrial morphology, which can regulate mitochondrial fission and fusion in mammalian cells,^20^ and serve as a predictive marker for the prognosis of patients with various diseases such as colorectal cancer and gallbladder cancer,^21^^,^^22^ can also be considered as a reference marker for T2DM. In addition, TTN is a gene encoding the giant skeletal muscle protein Titin,^23^ which is of great value in the study of tibial muscular dystrophy (TMD); while SMYD1 (the protein 1 gene of the SET and MYND domains) has an important role in sarcomere tissue. Crucial.^24^ Voltage-Dependent Anion Channel 1 (VDAC1) is one of the key proteins in regulating mitochondrial function, which is the main source of ATP for cell function, and they are also the center of cell signaling, cell division and apoptosis.^25^ In conclusion, the proteins regulated by the above key gene sets mainly play important roles in cell movement, insulin resistance, fatty acid metabolism, mitochondrial function regulation, and skeletal muscle. On the one hand, the result verifies the conjecture of this paper that T2MD is closely related to energy metabolism. On the other hand, it proposes the possibility of ATP5B and SLC27A4 as T2DM markers. Additionally, the reason why TTN is one of the key genes of T2DM in the study might be the truth that these sample tissues obtained from human skeletal muscle.

Furthermore, this paper uses the CIBERSORT algorithm to analyze the infiltration of immune cells, and compares the proportion of immune cells between T2DM patients and normal samples. The results show a significant difference in the proportion of immune cells between the two groups. In particular, compared with the control group, macrophages M2, CDB T-cells, monocytes and resting mast cells are highly expressed in T2DM group, while the percentage of T-cells, detected NK cells, naive B-cells, and naive CD4 T-cells are decreased. Studies have shown that M2 activity promotes cell proliferation and tissue repair, and has anti-inflammatory properties.^26^ The T-cells CDB plays a key role in preventing disease progression after Human Immunodeficiency Virus (HIV) infection.^27^

## Conclusion

In this study, a total of 19,217 DEGs and 269 DEGs were identified. T2DM-related signaling pathways and biological processes were identified through enrichment analysis, and the most enriched pathways were screened: thermogenesis, oxidative phosphorylation, chemical carcinogenesis-reactive oxygen species, prion disease, Parkinson's disease, and diabetic cardiomyopathy. Biological processes include three major components: cellular components, biological processes, and molecular functions, including collagen-containing extracellular matrix, mitochondrial protein complexes, mitochondrial inner membrane protein complexes and other cellular components; cellular respiration, aerobic respiration, organic Energy derivation of compound oxidation, biological processes such as ATP metabolism, extracellular matrix structural components, proton transport ATP synthase activity, rotation mechanism, redox-driven active transmembrane transporter activity and other molecular functions. PPI analysis screened 10 hub genes and provided possible roles of these hub genes in T2DM, but the relationship between hub genes and T2DM prediction requires further investigations. By immune infiltration analysis, differences in immune infiltration were found between the T2DM experimental group and normal controls. However, the specific relationship between hub genes and immune infiltration needs to be further explored to determine the role of the immune infiltration profile in the prognosis and treatment of T2DM.

## Declaration of competing interest

The authors declare no conflicts of interest.
